# The St. John's Ambulance Carriage

**Published:** 1887-10-01

**Authors:** 


### THE ST. JOHN'S AMBULANCE CARRIAGE.

A trained nurse thinks it may interest some readers to know that a patient of hers, a lady suffering from a disease, with a probability of hæmorrhage, was successfully removed from London to her country house (a distance of seventeen miles), in a St. John's ambulance carriage. The journey was accomplished in four hours and a half, without the patient experiencing any fatigue or inconvenience at the time, or suffering from ill-effects afterwards.

